# The importance of fine‐scale predictors of wild boar habitat use in an isolated population

**DOI:** 10.1002/ece3.9031

**Published:** 2022-06-22

**Authors:** Sonny A. Bacigalupo, Yu‐mei Chang, Linda K. Dixon, Simon Gubbins, Adam J. Kucharski, Julian A. Drewe

**Affiliations:** ^1^ Royal Veterinary College University of London Hatfield UK; ^2^ The Pirbright Institute Surrey UK; ^3^ London School of Hygiene & Tropical Medicine University of London London UK

**Keywords:** fine‐scale predictors, spatial logistic regression, species distribution, wild boar, wildlife habitat use, wildlife–livestock interface

## Abstract

1. Predicting the likelihood of wildlife presence at potential wildlife–livestock interfaces is challenging. These interfaces are usually relatively small geographical areas where landscapes show large variation over small distances. Models of wildlife distribution based on coarse data over wide geographical ranges may not be representative of these interfaces. High‐resolution data can help identify fine‐scale predictors of wildlife habitat use at a local scale and provide more accurate predictions of species habitat use. These data may be used to inform knowledge of interface risks, such as disease transmission between wildlife and livestock, or human–wildlife conflict.
2. This study uses fine‐scale habitat use data from wild boar (*Sus scrofa*) based on activity signs and direct field observations in and around the Forest of Dean in Gloucestershire, England. Spatial logistic regression models fitted using a variant of penalized quasi‐likelihood were used to identify habitat‐based and anthropogenic predictors of wild boar signs.
3. Our models showed that within the Forest of Dean, wild boar signs were more likely to be seen in spring, in forest‐type habitats, closer to the center of the forest and near litter bins. In the area surrounding the Forest of Dean, wild boar signs were more likely to be seen in forest‐type habitats and near recreational parks and less likely to be seen near livestock.
4. This approach shows that wild boar habitat use can be predicted using fine‐scale data over comparatively small areas and in human‐dominated landscapes, while taking account of the spatial correlation from other nearby fine‐scale data‐points. The methods we use could be applied to map habitat use of other wildlife species in similar landscapes, or of movement‐restricted, isolated, or fragmented wildlife populations.

## Graphical abstract

High‐resolution data can help identify fine scale predictors of wildlife habitat use at local scales, such as at wildlife‐livestock interfaces. This study uses fine‐scale presence data from wild boar (*Sus scrofa*) based on activity signs and direct field observations in a human‐dominated landscape. Spatial logistic regression models identified habitat‐based and anthropogenic predictors of wild boar signs, and the approach used could be applied to map the habitat use of other wildlife species in similar landscapes or of fragmented wildlife populations.

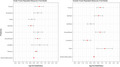

## 1 INTRODUCTION

Many infectious diseases pass between livestock and wildlife, in both directions. The westerly spread of African swine fever (affecting domestic pigs and wild boar, *Sus scrofa*) across Eastern Europe since 2007 (Sanchez‐Vizcaino et al., 2013) and the seasonal occurrence of avian influenza (affecting poultry and wild birds) (European Food Safety Authority et al., 2020) are two recent examples. Focus is usually placed on the risks to livestock from wildlife reservoirs of disease, with much less consideration given to the risks that livestock may pose to wildlife (Beauvais et al., 2019; Bozzuto et al., 2020; Wiethoelter et al., 2015). Wild boar are one of the most widely distributed mammals globally since being introduced in the 16th century onward to continents outside of their native Eurasia (Long, 2003; Massei et al., 2011; Massei & Genov, 2004). They are susceptible to diseases of major economic importance to the livestock industry such as African swine fever, foot‐and‐mouth disease, and bovine tuberculosis (Dixon et al., 2019; Grubman & Baxt, 2004; Naranjo et al., 2008). Monitoring wild boar distributions and sampling their populations are necessary in order to better understand, detect, manage, and even predict the occurrence of such diseases.

Monitoring elusive wildlife species such as wild boar is challenging owing to their wide geographic spread (Long, 2003), the resources and personnel needed (Engeman et al., 2013), their wariness of humans and limitations in the available methods of detection (Enetwild Consortium, Keuling et al., 2018). A variety of survey methods has been used to monitor wild boar abundance including tracking plots, dung counts, arial surveys, counts from vehicles, animal marking, hunting take rates, camera traps, and plot occupancy based on presence–absence observations (Engeman et al., 2013). While these methods aim to assess population size or estimate population trends in areas of known wild boar activity, it would also be useful to be able to estimate and predict the spatial distribution and abundance of wild boar in other areas where less is known about their occurrence (Vergne et al., 2020).

Identifying predictors of wild boar presence (or absence) can inform the development of models to predict current, and potentially future, wild boar distributions from global to regional scales (Enetwild Consortium, Croft et al., 2018; Rutten et al., 2019). Environmental predictors of wild boar presence such as habitat suitability, climate, topography, vegetation, and snow cover have been used to model wild boar distributions (Bosch et al., 2014; Vilaça et al., 2014), as well as anthropogenic predictors such as human disturbance (Croft et al., 2017). These predictions of wild boar distributions can be used to inform the development of transmission models for disease such as African swine fever virus (ASFV) and foot‐and‐mouth disease virus (FMDV) (Bosch et al., 2017; Croft et al., 2019; Croft, Massei et al., 2020) and to identify areas of spatial overlap between wild boar and domestic pigs where interspecies transmission could occur (Enetwild Consortium et al., 2020, 2021). Identifying areas of potential wildlife–livestock transmission (interfaces) could lead to more efficient disease surveillance, control, and prevention (Cross et al., 2019; Laguna et al., 2021). These data can be used along with livestock‐wild boar contact rates to model potential disease spill‐over events and evaluate the effectiveness of mitigation strategies (Manlove et al., 2019).

Predicting the presence or distribution of wildlife, such as wild boar, is often done over large distances which means generalizations are made based on broad‐scale data (e.g., many kilometers) (Enetwild Consortium, Croft et al., 2018), which may not reflect differences in habitat use over comparatively small areas. For example, in areas where wild boar enter areas of human activity and come into contact with people, large‐scale generalizations that wild boar avoid areas of human activity may not usefully inform local policy (Castillo‐Contreras et al., 2018; Dutton et al., 2015). Fine‐scale data have been used to more accurately identify areas of increased wildlife habitat use, particularly in areas where there are habitat variations over small distances, such as intertidal habitats (e.g., foraging preferences of oystercatchers, *Haematopus ostralegus* on seashores; Schwemmer et al., 2016) and in human‐modified landscapes (e.g., abundance of red foxes, *Vulpes vulpes* on farmland; Kammerle et al., 2018). Fine‐scale data allows for more accurate predictions about habitat use (Barbosa et al., 2010; Gastón & García‐Viñas, 2010; McPherson et al., 2006) and identifying predictors in areas near livestock could be used to inform knowledge of the risk of disease transmission from wild boar to livestock, and vice versa (Triguero‐Ocaña et al., 2021). One challenge of analyzing such fine‐scale data is how to control for spatial autocorrelation when sampling over increasingly smaller areas (Dormann, 2007; Legendre, 1993).

The aim of this study was to identify fine‐scale predictors for wildlife habitat use in areas with high levels of human activity where there is potential for wildlife contact with livestock, using a wild boar population the Forest of Dean in southwest England as an example. More broadly, the approach could be used to map habitat use of other wildlife species in similar landscapes, or movement‐restricted, isolated, or fragmented wildlife populations where broad‐scale data are not available or not representative. This information could be used in future to parameterize mathematical models of disease transmission within wildlife populations and between wildlife and livestock.

## 2 MATERIALS AND METHODS

### 2.1 Study area

**FIGURE 1:**
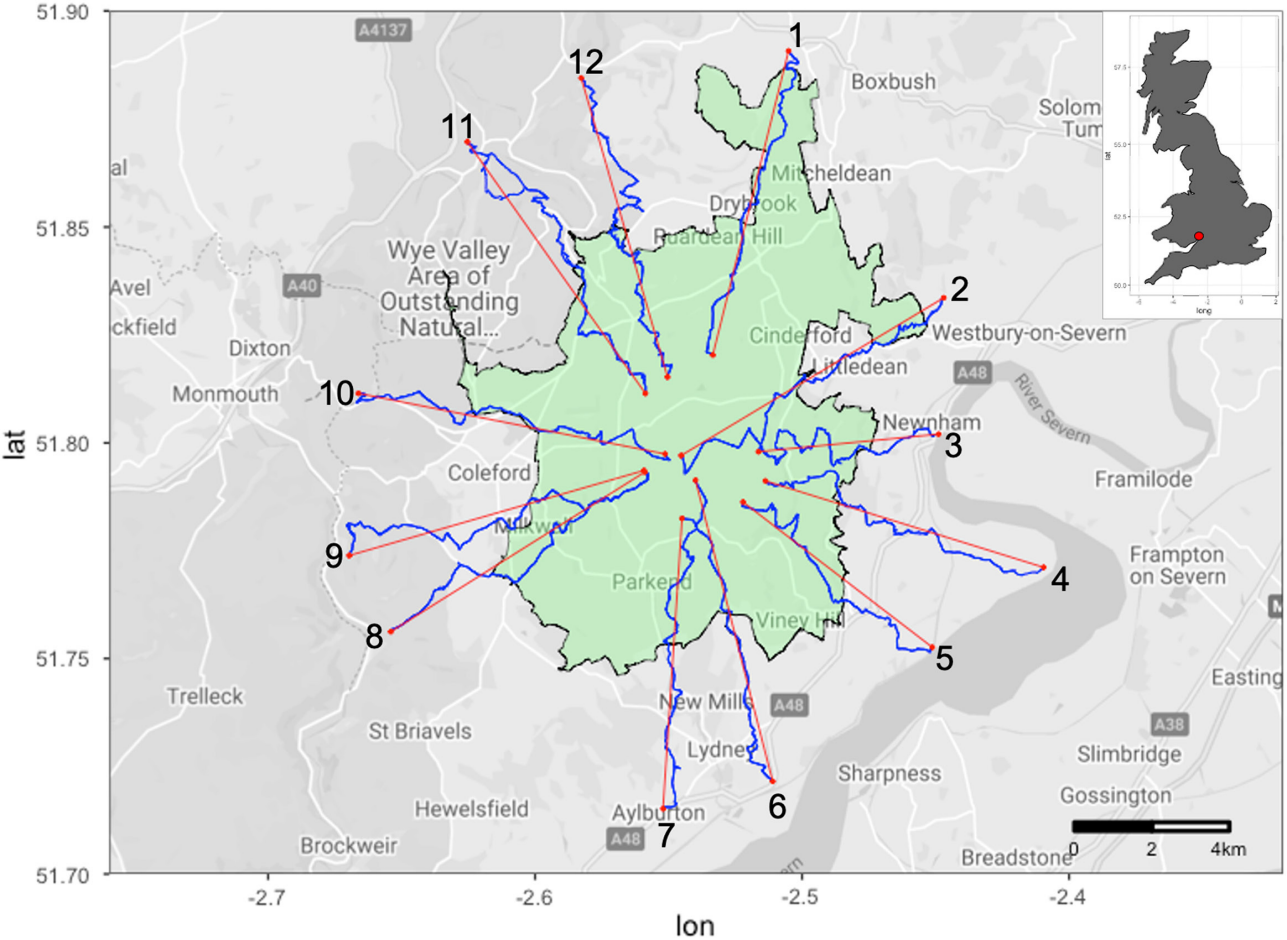
Map of the randomly selected line transects (red) and actual routes walked (blue) across the Forest of Dean (green). Transects started 3 to 4 km inside the forest boundary (black) and ended up to 5 km outside of it. Inset: Map of the location of the Forest of Dean (red circle) in Great Britain

This study was conducted in and around the Forest of Dean in Gloucestershire, United Kingdom, between the Severn and Wye rivers on the southern end of the English–Welsh border (Figure 1). The Forest of Dean is a popular tourist destination and hosts the largest population of wild boar in England, estimated at 1172 individuals (95% confidence interval: 885–1552) in 2019 (Gill & Waeber, 2019). The forest comprises 75 km^2^ of a mixture of broadleaved and coniferous woodland managed by Forestry England who also manage the wild boar population through year‐round culling. Sheep are free to roam freely within the Forest under Common Law. The surrounding landscape is predominantly arable, pastures and smaller woodlands and the decision to manage wild boar rests with individual landowners and local communities, and there is no requirement to control wild boar. This study covered an area encompassing the forest and extending to five kilometers outside the Forest of Dean statutory boundary (Figure 1).

### 2.2 Data collection

Twelve transect bearings radiating outward from the central point of the Forest of Dean (51.80°N, 2.53°W) were selected by first dividing the forest into 12 equal 30‐degree segments (from 000 to 029 degrees, 030 to 059 degrees, etc.), and then one bearing within each of these segments was randomly selected. This systematic approach ensured all regions of the forest were sampled. As it was not possible to walk straight along the exact bearing of each transect, public paths and roads that most closely followed each transect bearing were used. These were identified using the ViewRanger app (Augmentra Ltd, Cambridge, UK). Transects started from 3 km inside the statutory forest boundary and ended 5 km outside the boundary (Figure 1).

One transect per day was walked starting at sunrise, by the same person in October 2019 and again in March 2020. Location tracking in the ViewRanger app recorded regular GPS coordinates of the surveyor, the distance traveled and the time, automatically, allowing the route taken and the time at each location to be documented. While walking, paths, roads, and verges within approximately a 5‐m line of sight were scanned visually for wild boar signs. Locations of wild boar activity signs (footprints/tracks, rooting, rubbing, wallows, and boar sounds and sightings (Goulding, 2003)—see Table S1 for photos and descriptions) were recorded using the ViewRanger app on a mobile phone. Footprints were only included if they could be distinguished from other ungulates present in the forest (fallow deer (*Dama dama)*, roe deer (*Capreolus capreolus*), muntjac (*Mutiacus reevesi*), and domestic sheep (*Ovis aries*)) as described in Table S1. Potential predictors of wild boar habitat use relating to habitat and human activity were recorded in the same way, regardless of whether wild boar signs were present or not—see Table S2 for predictors and definitions. The start and end locations of activity signs and predictors were recorded where they covered large areas, either in the Viewranger app or in voice recordings, and the time that these voice recordings were made was used to determine the location along the transect. Detailed information on the recording protocol can be found in Tables S1 and S2.

### 2.3 Data analysis

Straight‐line distances between all recorded locations (of habitats, features, and wild boar signs) and the center of the forest were calculated. Data were then collated into 50 m segments for analysis (i.e., 50 m‐spaced concentric rings emanating from the center of the Forest). Autumn transects were split into 1763 segments (744 inside the forest boundary; 1019 outside the forest boundary) while Spring transects were split into 1749 segments (740 inside the forest boundary; 1009 outside the forest boundary), due to locations not being recorded in four segments in Spring and three segments in Autumn, and there being 13 flooded segments at the end of transect 4 in Spring. Where the walking routes overlapped, for example, in transects 8 and 9, data from only one transect were used for these parts, decided randomly by a coin toss.

Spatial autocorrelation between the residuals of logistic regression models for all subsets of data was assessed with Moran's I test statistic in the DHARMa package (version 0.3.1) in (Hartig & Hartig, 2020; Kelejian & Prucha, 2001; Moran, 1950) in R (version 4.0.0) (R Core Team, 2020).

Initial exploratory analyses of potential predictors of wild boar activity signs were done using univariable spatial logistic regression, using the SPAMM package (version 3.3.0) in R (Rousset, 2020). Odds ratios (OR) and 95% confidence intervals (CI) were reported. All variables were included in the final multivariable analysis regardless of performance in the univariable models. Predictors for the presence of wild boar activity signs were identified through backward stepwise multivariable spatial logistic regression with repeated measures, using the SPAMM package (version 3.3.0). Best performing multivariable models were selected based on the inclusion of predictors with a *p*‐value < .2 and by AIC due to the large number of sample points in the models. Odds ratios (OR) and 95% confidence intervals (CI) were reported. Data collected inside and outside the statutory forest boundary were analyzed separately due to the variation in habitat types and other predictors between the two regions. For example, some predictors such as crops were absent inside the Forest, and because of differences in management of wild boar between these areas. Spatial autocorrelation was accounted for by including a Matérn covariance function as a random effect (Rousset & Ferdy, 2014). Residual diagnostics of the spatial logistic regression models were visually inspected for outliers and evaluated using DHARMA scaled residual plots. Multi‐collinearity of model predictors was informed by VIF using the Performance package in R (version 0.9.0).

## 3 RESULTS

### 3.1 Wild boar activity signs and predictors recorded

**FIGURE 2:**
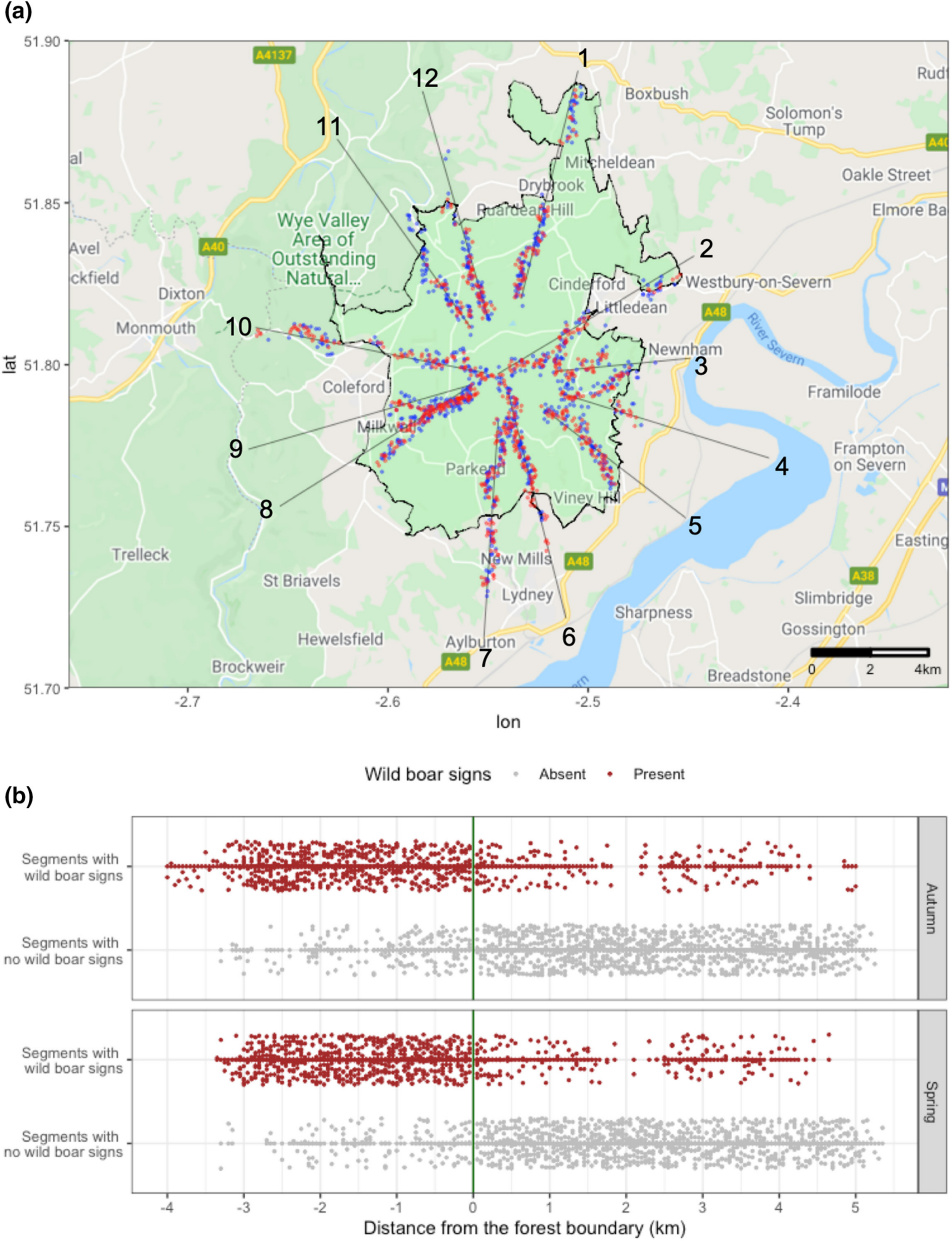
Presence of wild boar signs inside and outside the Forest of Dean. (a) Distribution of wild boar activity signs along transects (dark gray) in autumn (red) and spring (blue) with jitter; (b) Distribution of 50 m‐segments with (red) and without (gray) wild boar activity signs in terms of distance from the boundary of the forest (green line), in autumn and spring, with vertical jitter

In Autumn, signs of wild boar activity were observed in 561/744 (75%) of transect segments inside the forest boundary, and 132/1019 (13%) of transect segments outside the forest boundary. In Spring, signs of wild boar activity were observed in 615/740 (83%) of transect segments inside the forest boundary, and 132/1009 (13%) of transect segments outside the forest boundary (Figure 2). Rooting was the most commonly recorded sign and was present in 97% (1400/1440) of the total segments with wild boar activity, followed by footprints (19%; 279/1440), wallows (3%; 43/1440), fence damage (0.8%; 11/1440), sightings (0.8%; 8/1440), tree/post rubbing (0.4%; 6/1440), and feces (0.1%; 2/1440). Wild boars were directly sighted on four occasions in Autumn 2019 and four occasions in Spring 2020.

Predictors recorded in Spring and Autumn are shown in Table 1. The most frequently recorded habitat type was forest (52% and 51% of segments in Autumn and Spring, respectively) followed by field (37% and 36%) and residential (16% and 17%). Tarmac roads (44% and 48%) and dirt paths (26% and 27%) were the most common track types. All predictors showed considerable differences in frequency inside and outside the statutory forest boundary, notably livestock were recorded in a total of 15 segments inside the forest boundary compared to 234 segments outside, and crop fields were only recorded in segments outside the forest boundary in both seasons.

**TABLE 1:** Number of transect segments containing potential predictors of wild boar habitat use, stratified by season and whether inside or outside the statutory boundary of the Forest of Dean

Predictor	Autumn			Spring		
	Inside forest	Outside forest	Total (%)	Inside forest	Outside forest	Total (%)
Habitat						
Forest	717	208	925 (52)	711	182	893 (51)
Scrubland	51	21	72 (4)	35	9	44 (3)
Residential	29	250	279 (16)	32	272	304 (17)
Field	28	632	660 (37)	29	608	637 (36)
Other habitat	6	38	44 (2)	6	37	43 (2)
Track						
No path	54	208	262 (15)	31	186	217 (12)
Dirt path	302	160	462 (26)	333	136	469 (27)
Dirt road	58	24	82 (5)	39	29	68 (4)
Gravel path	40	23	63 (4)	49	24	73 (4)
Gravel road	171	110	281 (16)	148	114	262 (15)
Tarmac path	10	10	20 (1)	14	10	24 (1)
Tarmac road	203	581	784 (44)	221	613	834 (48)
Feature						
Livestock	9	147	156 (9)	6	87	93 (5)
Water	25	5	30 (2)	16	6	22 (1)
Crops	0	80	80 (5)	0	25	25 (1)
Litter bin	3	18	21 (1)	3	21	24 (1)
Park	3	13	16 (1)	2	13	15 (1)
Building outside of a residential habitat	54	166	220 (12)	45	160	205 (12)

**FIGURE 3:**
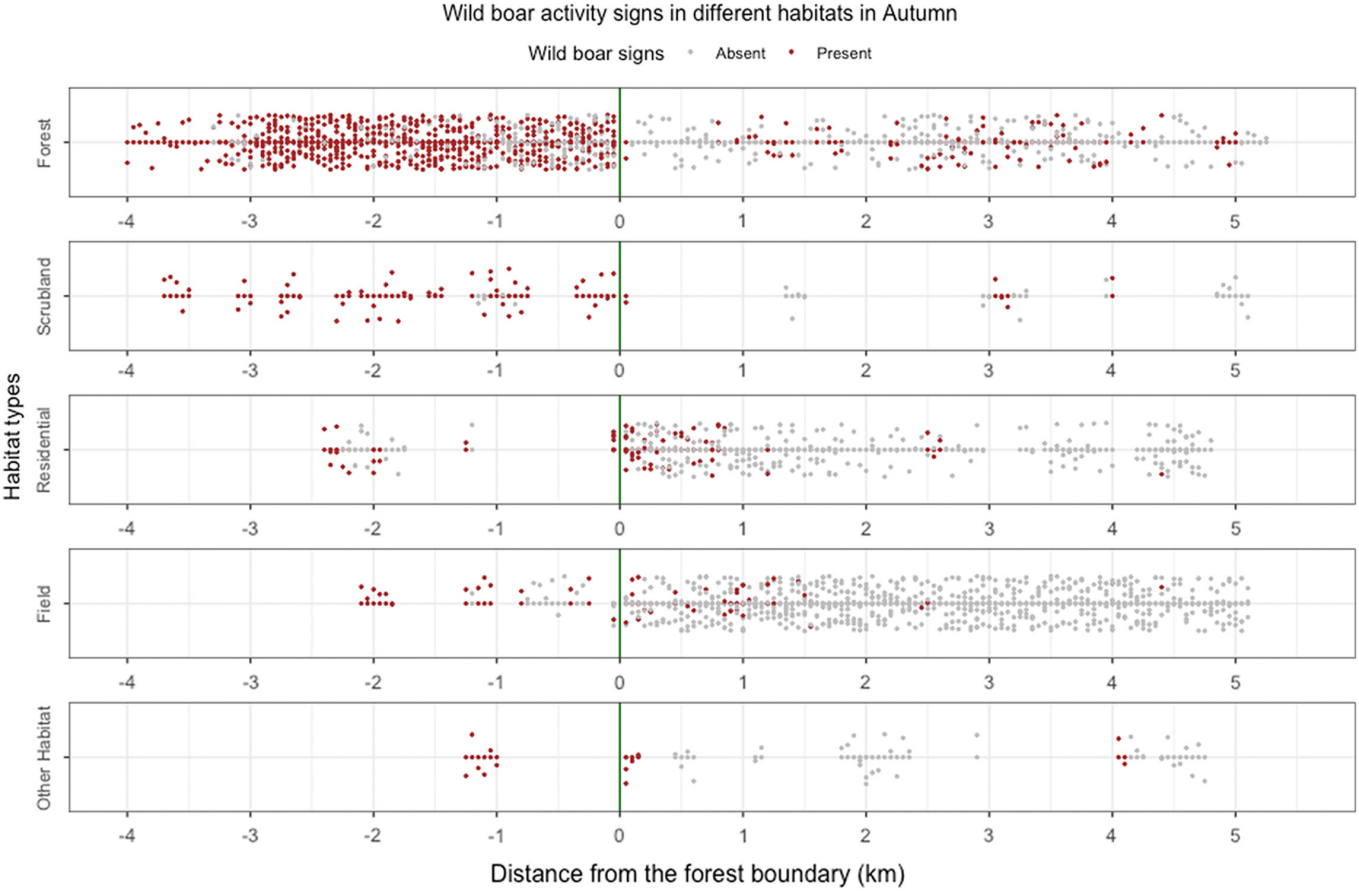
Wild boar signs by habitat type. Distribution of 50 m‐segments with (red) and without (gray) wild boar activity signs in each type of habitat in autumn, in terms of distance from the boundary of the forest (green line)

The segments inside the statutory forest boundary containing wild boar activity signs featured forest and scrubland habitats (Figure 3). The majority of the segments with wild boar activity signs outside the forest boundary also featured forest habitats, although wild boar signs were also recorded in field and residential habitats within 2 km of the forest boundary (Figure 3). Segments with wild boar activity signs more than 2 km from the forest boundary were recorded in two north‐eastern transects that re‐entered the statutory Forest of Dean (Transects 1 and 2), and in privately owned forested areas to the south and west of the Forest of Dean (Transects 6 and 7) (Figures 2 and 3).

### 3.2 Spatial logistic regression analysis

Moran's *I* test statistic showed significant spatial autocorrelation between the residuals of multivariable logistic regression models for all subsets of data. Spatial correlation was close to zero when (a) distances between locations approached 1 km inside the forest, and (b) distances between locations approached 5 km outside the forest (Figure S1).

Initial exploratory univariable analysis using all data suggested wild boar signs were more likely to be observed in spring compared to autumn (OR: 1.45; 95% CI: 1.15, 1.83). Univariable models for spring and autumn separately showed wild boar signs were more likely to be observed inside than outside the forest boundary in both seasons of the study (autumn OR: 5.74; 95% CI: 1.30, 25.34; spring OR: 6.32; 95% CI: 1.84, 21.61). Data were therefore separated by both season and by whether observations occurred inside or outside the forest boundary. Comparing the predictors and their effect sizes between univariable and multivariable models (Figures S2 and S3) indicated reasonable consistency between seasons and more variability for inside and outside the forest boundary. The marked variation between the presence of wild boar signs, habitat types, and the presence of different predictors inside and outside the forest boundary meant that many interactions between the forest boundary and other predictors would need to be accounted for. Therefore, the forest boundary was not included as a variable and two separate spatial repeated measures logistic regression models, for wild boar signs inside and outside the forest boundary, were developed.

**FIGURE 4:**
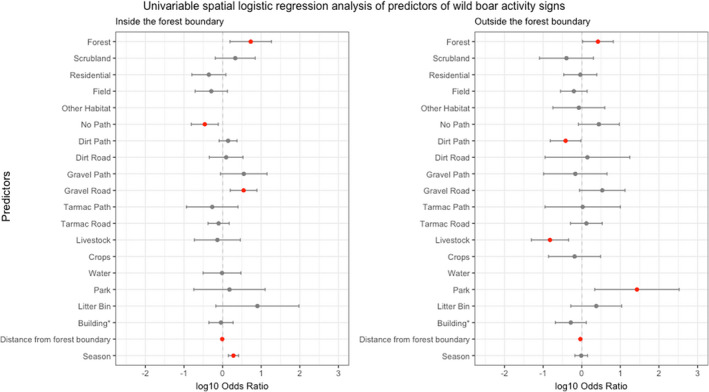
Univariable spatial analysis results (log odds ratios and 95% confidence intervals) for predictors of wild boar activity signs inside and outside the forest boundary, estimated using a repeated measures model. Predictors with *p* < .05 are shown in red. Data are unavailable for three predictors where numerical issues arose due to separation among the sample points

Further exploratory univariable spatial logistic regression analyses of data stratified by forest boundary (Figure 4 and Table 2) show that wild boar signs were more likely to be seen inside the forest boundary in Spring (OR: 1.91; 95% CI: 1.41, 2.59) but season was not a significant predictor of wild boar signs outside the forest boundary. Wild boar signs were more likely to be seen in forested segments both inside (OR: 5.35; 95% CI: 1.55, 18.46) and outside the statutory forest boundary (OR: 2.63; 95% CI: 1.06, 6.57). Distance from the forest boundary was allocated a negative value inside the forest, zero at the boundary, and a positive value outside of the forest. Thus, the lowest value (−4 km) occurred at the center of the forest near where the transects started, and the highest value (5 km) occurred outside the forest at the end of the transects (e.g., Figure 2b). Inside the forest, wild boar signs were less likely to be seen as distance from the center of the forest increased (OR: 0.98 per 50 m distance moved from center), and outside the forest they were less likely to be recorded further away from the forest boundary (OR: 0.92 per 50 m distance). Inside the forest, wild boar signs were more likely to be seen in segments with gravel roads, and less likely to be seen in segments with no path or road present. Outside the forest, wild boar signs were more likely to be recorded in segments with recreational parks and were less likely to be recorded in segments with livestock and dirt paths.

**TABLE 2:** Univariable spatial analysis showing odds ratios and 95% confidence intervals for predictors of wild boar activity signs inside and outside the forest using a repeated measures model

	Predictor	Inside forest		Outside forest	
		Odds ratio (95% CI)	*p* Value	Odds ratio (95% CI)	*p* Value
Habitat	**Forest**	**5.35 (1.55, 18.46)**	**<.01**	**2.63 (1.06, 6.57)**	**.04**
	Scrubland	2.13 (0.65, 7.01)	.21	0.40 (0.08, 2.00)	.27
	Residential	0.44 (0.16, 1.22)	.11	0.92 (0.34, 2.46)	.87
	Field	0.51 (0.19, 1.33)	.17	0.62 (0.28, 1.38)	.24
	Other habitat			0.84 (0.18, 3.96)	.83
Track	**No path**	**0.35 (0.15, 0.77)**	**.01**	2.77 (0.82, 9.39)	.10
	**Dirt path**	1.39 (0.81, 2.36)	.23	**0.38 (0.15, 0.95)**	**.04**
	Dirt road	1.24 (0.45, 3.38)	.68	1.40 (0.11, 17.56)	.79
	Gravel path	3.54 (0.88, 14.22)	.07	0.68 (0.10, 4.52)	.69
	**Gravel road**	**3.50 (1.58, 7.76)**	**<.01**	3.40 (0.88, 13.22)	.08
	Tarmac path	0.54 (0.12, 2.50)	.43	1.06 (0.11, 10.01)	.96
	Tarmac road	0.79 (0.42, 1.48)	.46	1.32 (0.51, 3.40)	.57
Feature	**Livestock**	0.73 (0.19, 2.88)	.65	**0.15 (0.05, 0.46)**	**<.001**
	Crops			0.65 (0.14, 3.09)	.59
	Water	0.97 (0.31, 2.99)	.95		
	Litter bin	7.97 (0.67, 95.09)	.10	2.37 (0.52, 10.79)	.26
	**Park**	1.51 (0.18, 12.64)	.71	**26.93 (2.17, 333.81)**	**.01**
	Building^a^	0.91 (0.44, 1.87)	.80	0.52 (0.21, 1.31)	.17
	**Distance from forest boundary**	**0.98 (0.96, 0.99)**	**<.01**	**0.92 (0.87, 0.98)**	**.01**
	**Season**	**1.91 (1.41, 2.59)**	**<.001**	0.97 (0.66, 1.41)	.87

*Note*: Predictors with *p* < .05 are shown in bold. Data are missing for some predictors that caused numerical issues due to separation among the sample points (shaded gray boxes).

^a^ Specifically, a building located outside of a residential habitat.

**FIGURE 5:**
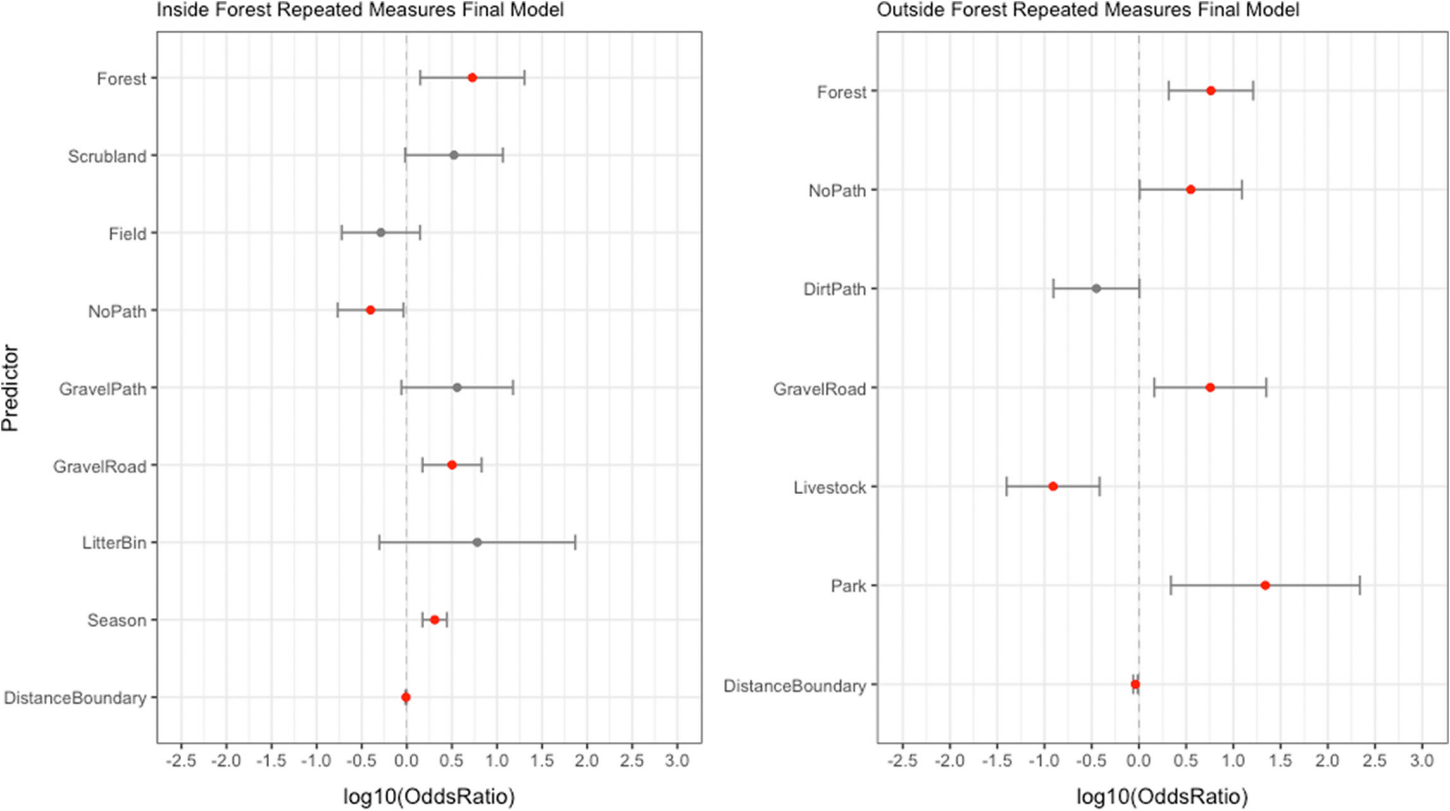
Multivariable spatial analysis results (log odds ratios and 95% confidence intervals) for predictors of wild boar activity signs with *p*‐values‐values < .2 inside and outside the forest boundary, estimated using a repeated measures model. Predictors with *p* < .05 are shown in red

Final multivariable analyses (Figure 5 and Table 3) revealed that inside the forest boundary, there were increased odds of wild boar activity signs in Spring compared to Autumn (OR: 2.04; 95% CI: 1.49, 2.78) but season did not appear in the model for outside the forest. Forest habitat was a significant predictor of wild boar signs both inside (OR 5.33; 95% CI: 1.41, 20.17) and outside the statutory forest boundary (OR: 5.79; 95% CI: 2.07, 16.21). Inside the forest boundary, wild boar signs were more likely to be seen in transect segments containing litter bins (OR: 2.04; 95% CI: 0.50, 73.79). Outside the forest boundary, wild boar signs were less likely to be seen in segments where livestock were recorded (OR: 0.12; 95% CI: 0.04, 0.38), but more likely to be seen in segments featuring parks (OR: 21.83; 95% CI: 2.18, 218.43). Aside from the comparing the number of wild boar signs inside and outside the forest boundary, the size of the differences in habitat preferences between the two regions were difficult to quantify since some predictors that remained in the final model for inside the forest boundary were absent from the model for outside the forest boundary, and vice versa, and predictors that remained in both models appeared to overlap in their confidence intervals.

**TABLE 3:** Multivariable spatial analysis showing odds ratios and 95% confidence intervals for predictors of wild boar activity signs with *p*‐values < .2 inside and outside the forest using a repeated measures model

	Predictor	Inside forest		Outside forest	
		Odds ratio (95% CI)	*p* Value	Odds ratio (95% CI)	*p* Value
Habitat	**Forest**	**5.33 (1.41, 20.17)**	**.01**	**5.79 (2.07, 16.21)**	**<.001**
	Scrubland	3.33 (0.96, 11.61)	.06		
	Field	0.52 (0.19, 1.40)	.19		
Track	**No path**	**0.40 (0.17, 0.92)**	**.03**	**3.54 (1.02, 12.33)**	**.05**
	Dirt path			0.35 (0.12, 1.01)	.05
	Gravel path	3.62 (0.87, 15.05)	.08		
	**Gravel road**	**3.17 (1.49, 6.74)**	**<.01**	**5.70 (1.45, 22.30)**	**.01**
Feature	**Livestock**			**0.12 (0.04, 0.38)**	**<.001**
	Litter bin	6.05 (0.50, 73.79)	.16		
	**Park**			**21.83 (2.18, 218.43)**	**<.01**
	**Distance from forest boundary**	**0.98 (0.97, 0.99)**	**<.01**	**0.92 (0.87, 0.97)**	**<.01**
	**Season**	**2.04 (1.49, 2.78)**	**<.001**		

*Note*: Predictors with *p* < .05 in the final model are shown in bold. Data are missing for predictors with p >0.2 that were excluded from the model (shaded gray boxes).

The residuals of both multivariable spatial logistic regression models were standardized to values between 0 and 1 (Figure S4). Residuals appeared randomly distributed with no obvious dispersion in the Q‐Q plots and good agreement between observed and expected values. There was low collinearity between predictors for both models.

## 4 DISCUSSION

### 4.1 The need for fine‐scale data of species habitat use

The logistic regression models developed in this study are based on fine‐scale observations of wild boar field signs to infer habitat use data at a 50‐m resolution. Other models have used environmental factors to predict wild boar data sampled over comparatively larger areas, for example, regional models developed for regions of Spain and Japan used wild boar presence–absence data at around 150 and 25 km^2^ resolutions, respectively (Acevedo et al., 2011; Honda, 2009). This more coarse data is useful where environmental conditions such as climate and topography vary over large distances, but for isolated populations mostly confined to a relatively small region, or where the area of interest is a comparatively small geographical range such as at human–wildlife interfaces, fine‐scale data may be more suitable since some environmental factors may be fairly homogenous across the area and wildlife may behave differently in these areas. Furthermore, in areas with high levels of human activity, habitat suitability and anthropogenic features show strong variation over small distances, which can only be accounted for by finer‐scale data. Wild boar in the Forest of Dean are in close proximity to areas of human activity and enter surrounding towns and villages to forage (Dutton et al., 2015), and this study identified signs of wild boar habitat use in these areas (Figure 3). Therefore, wild boar behavior in human‐dominated landscapes may not be representative of behaviors seen in other wild boar populations or predicted in other models. Our finding that forest‐type habitat is a consistently significant predictor of wild boar habitat use, agrees with findings of other distribution models (Enetwild Consortium, Croft et al., 2018). However, by using fine‐scale field observations along transects, this study has shown that additional predictors of wild boar habitat use can be identified that may be particular to human‐dominated landscapes, such as livestock, litter bins, gravel paths and roads, and recreational parks. Findings such as these could help target further monitoring of wild boar populations in areas surrounding the Forest of Dean and in other places where wild boar occur in areas of high human activity and could inform more precise models of wild boar habitat use across these regions.

### 4.2 Informing further wild boar monitoring

Wild boar in the Forest of Dean are currently a relatively isolated population due to being surrounded by main roads, the rivers Severn and Wye, and there is anecdotal evidence of private hunting pressure in areas surrounding the forest (Croft, Franzetti et al., 2020). This relative isolation means that wild boar densities in the Forest of Dean are high; 15 per km^2^ compared with European populations density where estimates over 2 per km^2^ could be considered high (Gill & Waeber, 2019; Pittiglio et al., 2018). This, as well as culling pressure from Forestry England, might encourage wild boar to disperse and their distribution to expand, and there are numerous areas outside the Forest of Dean that could form suitable wild boar habitat. Such areas could be surveyed to monitor this expansion by looking for predictors of habitat use, for example, forest‐type habitat and areas close to forest boundaries, and areas with recreational parks and litter bins. Surveying forest‐type areas in Spring may increase the likelihood of detecting wild boar presence in new areas. Additionally, predictors of decreased likelihood of wild boar habitat use could be surveyed less or not at all, such as areas inside forests with no paths.

The spatial analysis incorporated into these models revealed correlation between locations was near zero when approaching distances of 1 km inside the Forest of Dean boundary, and at distances of 5 km outside the forest boundary. This suggests that monitoring wild boar using transects spaced these distances apart could achieve an adequate resolution of the likelihood of wild boar habitat use in that area (and that closer transects are unnecessary). Understanding where likely areas of wild boar expansion are likely to be, and confirming wild boar habitat use in those areas, is an important step in order to assess the disease risk to and from livestock.

### 4.3 Informing wild boar distribution models

Fine‐scale data are needed to model wildlife habitat use in areas with strong environmental gradients (Schwemmer et al., 2016) and is important in the Forest of Dean due to the variation in landscape over relatively short distances, the limited distribution of wild boar, and the high levels of human activity and anthropogenic features. We have shown that fine‐scale data can be useful to identify predictors of wild boar habitat use in this area, and the effects of these predictors could be incorporated into wild boar distribution models. The incorporation of models based on fine‐scale data into existing broad‐scale wild boar distribution models may increase their precision and go some way to addressing the uncertainty that is seen where there is strong landscape variation on local scales (McInerny & Purves, 2011). Our finding that anthropogenic features such as litter bins, parks, and livestock could be important predictors of wild boar activity in human‐dominated landscapes could be used to model wild boar habitat use at the interface where human activity encroaches into wild boar habitat. This information could be further used to parameterize models of disease transmission between wild boar and livestock. However, collection of such high‐resolution data and fine‐scale habitat use data is time‐consuming and so focusing the collection of this data to areas of high broad‐scale model uncertainty, and the continued use of coarse data to model wild boar distributions across large regions, would be necessary to increase efficiency.

This study has some limitations. The use of transects and field observations mean data were recorded at only a snapshot in time, and repeated studies would be needed to identify variations over longer time periods. Wild boar activity signs indicate only habitat use of wild boar, not non‐use which is important to establish when monitoring wild boar in new areas of introduction; a lack of activity signs does not necessarily mean a lack of wild boar, particularly in areas where signs may be hard to detect, such as on tarmac in residential areas. In these areas, it may be appropriate to collect presence data in other ways such as by direct observation, public sightings, or camera traps.

### 4.4 Applications of fine‐scale habitat use data to other systems

The approach used in this study is not limited to wild boar populations. It is applicable to other systems where fine‐scale data may more precisely represent wildlife distributions on a local scale, such as movement‐restricted, isolated, or fragmented populations. Potential systems include small populations concentrated over smaller distances, for example, endangered populations of babirusa (*Babyrousa* spp.) occupying smaller and smaller habitats (Macdonald, 2017), or populations over small land areas such as invasive alien species on islands (Russell et al., 2017), or species where small numbers have been introduced and have not yet expanded such as Eurasian beaver reintroduction to parts of Europe (Smeraldo et al., 2017). Importantly, they are applicable to other interfaces where wildlife and human activities overlap, in particular around livestock farming. While the methods used here are more suitable for wildlife species and habitat types for which detection by direct sightings or indirect field activity signs is favorable, habitat use data collected by other means such as camera traps or arial surveys, or global positioning system collars, could also be used to identify predictors of habitat use, as long as data are collected on a sufficiently fine‐scale relative to the study area of interest.

As human activity and particularly livestock farming further expands into wildlife habitats, predicting the presence of wildlife species such as wild boar will become increasingly important to target population monitoring and disease surveillance efforts. This study demonstrates techniques to establish predictors of wildlife habitat use at a fine resolution using wild boar as an example. These methods could be applied to map habitat use of other wildlife species in similar landscapes, or movement‐restricted, isolated, or fragmented wildlife populations where broad‐scale data are not available or not representative.

## AUTHOR CONTRIBUTIONS

**Sonny A. Bacigalupo:** Conceptualization (equal); data curation (lead); formal analysis (lead); investigation (lead); methodology (equal); project administration (lead); writing – original draft (lead); writing – review and editing (equal). **Yu‐mei Chang:** Formal analysis (supporting); writing – review and editing (equal). **Linda K. Dixon:** Conceptualization (equal); methodology (equal); writing – review and editing (equal). **Simon Gubbins:** Conceptualization (equal); methodology (equal); writing – review and editing (equal). **Adam J. Kucharski:** Conceptualization (equal); methodology (equal); writing – review and editing (equal). **Julian A. Drewe:** Conceptualization (equal); funding acquisition (lead); methodology (equal); project administration (supporting); supervision (lead); writing – original draft (supporting); writing – review and editing (equal).

## CONFLICT OF INTEREST

The authors declare there are no competing interests.

## Supporting information


Figure S1

Figure S2

Figure S3

Figure S4

Table S1

## Data Availability

The dataset used in this analysis is archived on the Dryad Digital Repository https://doi.org/10.5061/dryad.4mw6m90cx.
