# Supplementary figures and images for: The importance of fine‐scale predictors of wild boar habitat use in an isolated population
Source: Ecol Evol. 2022 Jun 22;12(6):e9031. doi: 10.1002/ece3.9031 (PMC9217887; doi:10.1002/ece3.9031)

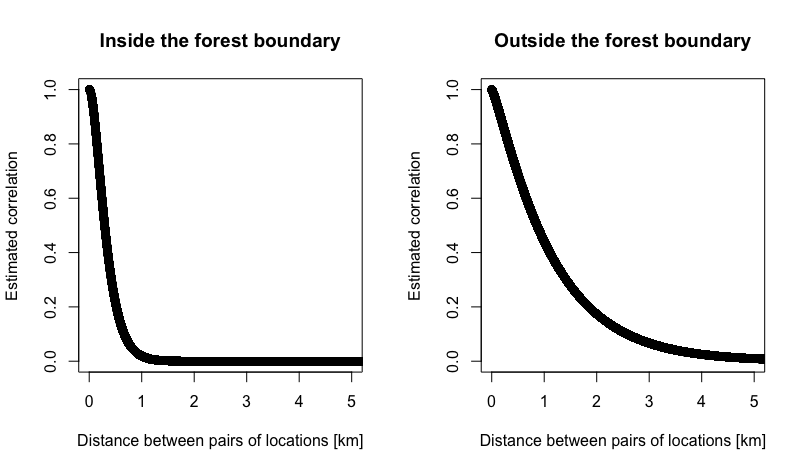

Supplement: Supplementary file 1 — Figure S1 [file ECE3-12-e9031-s002.tiff]

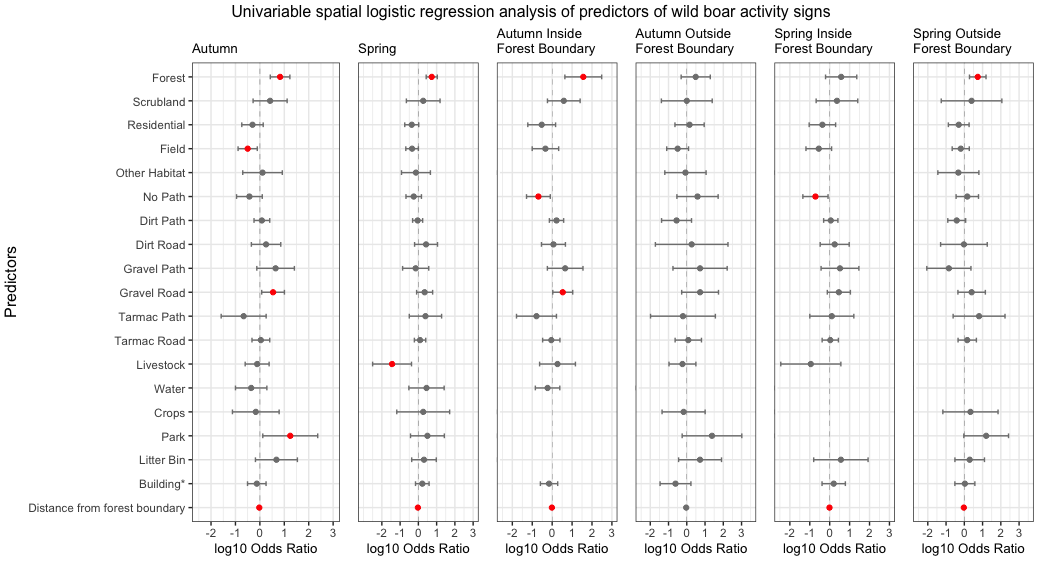

Supplement: Supplementary file 2 — Figure S2 [file ECE3-12-e9031-s001.tiff]

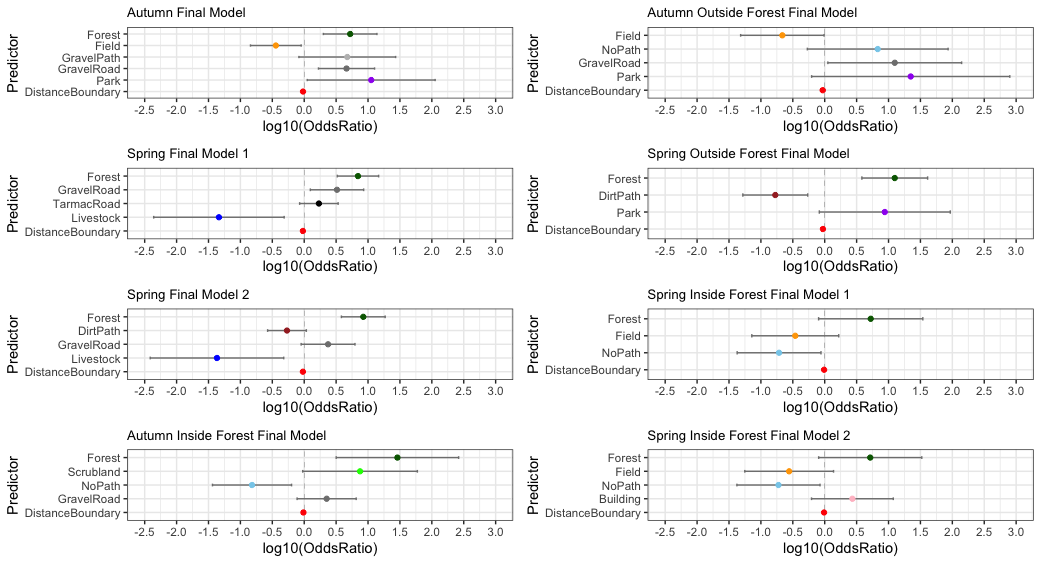

Supplement: Supplementary file 3 — Figure S3 [file ECE3-12-e9031-s005.tiff]

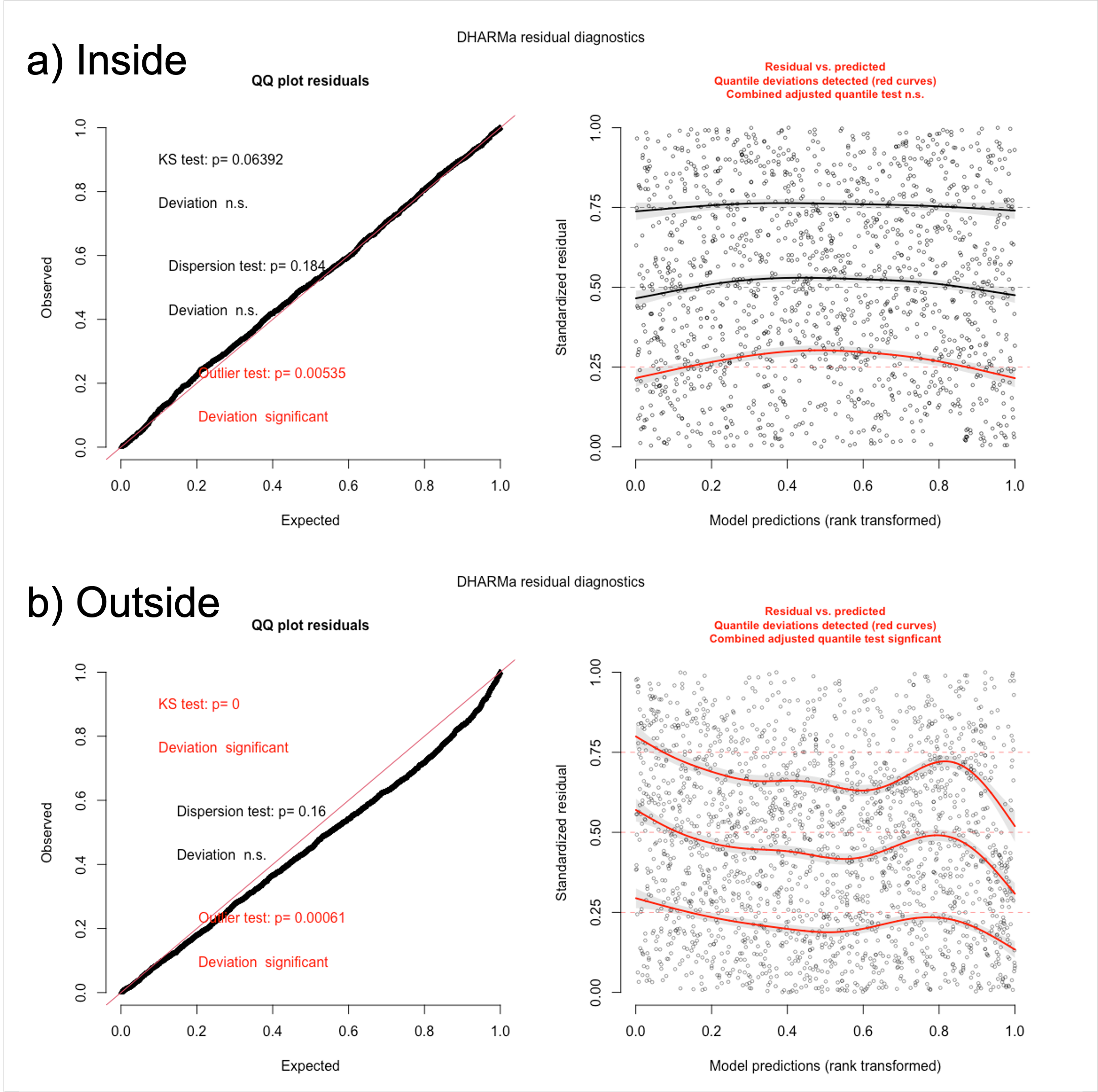

Supplement: Supplementary file 4 — Figure S4 [file ECE3-12-e9031-s003.tiff]
