# Supplementary material for: The importance of fine‐scale predictors of wild boar habitat use in an isolated population
Source: Ecol Evol. 2022 Jun 22;12(6):e9031. doi: 10.1002/ece3.9031 (PMC9217887; doi:10.1002/ece3.9031)
Supplement: Supplementary file 5 — Table S1 [file ECE3-12-e9031-s004.docx]

Table S1: Descriptions and photographs of the wild boar activity signs recorded.

| Field sign | Description | Example |
| --- | --- | --- |
| Rooting | Areas of disturbed earth distinguishable from badger foraging or similar activity by large intact chunks of overturned earth or the presence of wild boar footprints. Discounted if there is evidence of machine involvement such as tyre tracks. | 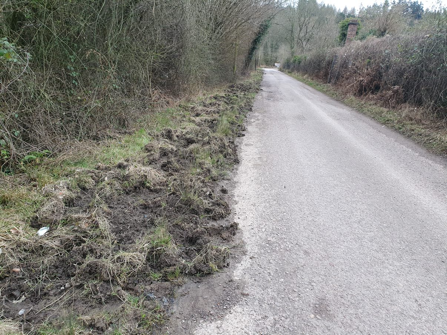 |
| Tracks | Hoofed claw marks distinguishable from deer and sheep by rounded claws and blunt toes as opposed to pointed toes, and by the wide set position of dew claws towards the outside of the hoof print rather than directly behind the heel. | 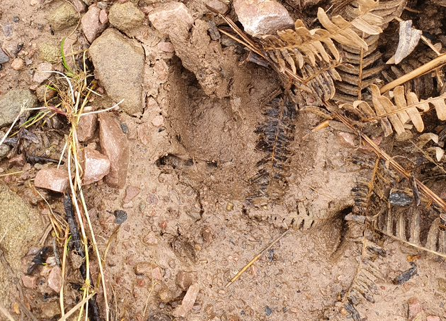 |
| Wallow | A small body of water that is larger than a wild boar, with accompanying wild boar footprints or other wild boar signs. | 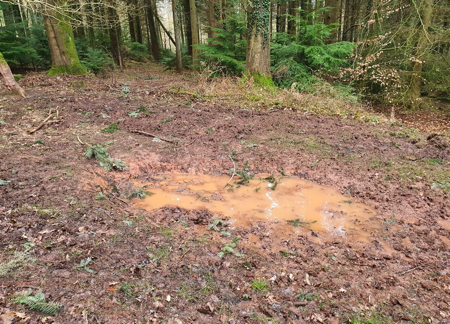 |
| Rubbing | A tree or post with bark rubbed away at the base with or without wild boar hair embedded. | 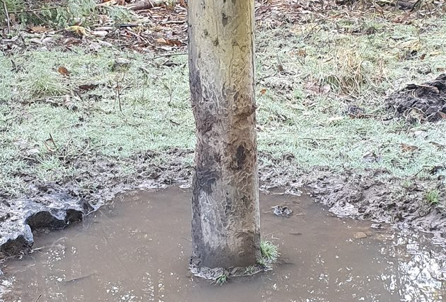 |
| Sighting | The direct visualisation of a wild boar, or the sound of wild boar grunting or squealing in nearby undergrowth. | 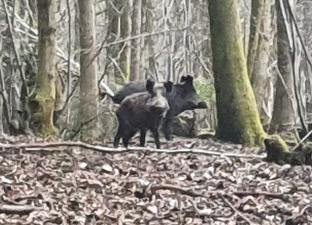 |

The start and end locations of rooting signs were recorded in instances where rooting was present for more than five metres, and where they were absent for more than five metres, respectively. The surrounding areas were also visually scanned for wild boar and rubbed tree bark, and wild boar sounds such as grunts and squeals were listened for. Areas of rooting, wallows and muddy ground suitable for footprints were favoured while walking along paths and roads, and were inspected for less obvious wild boar signs, such as footprints and faeces. The Viewranger app recorded the distance travelled in real time, and once a wild boar footprint was identified, footprints were then not looked for until the next fifty metre milestone was reached: for example, if a footprint was recorded after walking 315 metres, no more footprints would be actively looked for until a further 35 metres (350 metres total) had been walked. The start and end locations of footprints were recorded, as were locations when no footprints were seen for fifty metres. Potential predictors of wild boar habitat use relating to habitat and human activity were also recorded (Table S2) either in the Viewranger app when they covered small areas e.g., litter bins and stand-alone buildings, or in a voice recording when documenting the start and end points of predictors that covered large areas such as when entering and leaving a field or residential area. The time that these voice recordings were made was used to determine the location along the transect.

Table S2: Definitions of the predictors of wild boar activity identified during field observations in the Forest of Dean in 2019-2020, refined to avoid collinearity between variables.

| **Category** | **Predictor** | **Definition** |
| --- | --- | --- |
| **Habitats** | Forest | An area surrounded by trees with few, if any, buildings. |
|  | Scrubland | An area of dense vegetation above 1m tall. |
|  | Residential | An area characterised by a high density of dwellings, tarmac roads and pavements and street lighting. |
|  | Field | An open area of land bounded by a fence. |
|  | Other habitat | A habitat that is none of the above. |
| **Track Type** | Paths | A track or route not wide enough for a vehicle, and usually marked as a footpath or bridleway on an Ordnance Survey map. Paths were categorised as: dirt (non-paved road with no stone chippings or gravel), gravel (non-paved road with stone chippings or gravel) or tarmac (paved road). |
|  | Roads | A track or route wide enough for a vehicle to pass easily, and usually marked as a road on an Ordnance Survey map. Roads were also categorised as: dirt, gravel or tarmac as above. |
| **Features** | Livestock | Presence of livestock in the immediate area. |
|  | Water | Presence of a permanent body of water, either still or flowing. |
|  | Crops | Presence of crops planted systematically in a field. |
|  | Litter Bin | Presence of a litter bin. |
|  | Park | Presence of a recreational park. |
|  | Building in non-residential habitat | The presence of any building that is not in a residential habitat, for example a dwelling on a dirt track in the forest. |
|  | Distance from forest boundary | Distance from the outside boundary of the Forest of Dean. A negative value indicates a location inside the forest, and a positive value indicates a location outside the forest. |
